# Multimodal Brain Tumor Classification Using Convolutional Tumnet Architecture

**DOI:** 10.1155/2024/4678554

**Published:** 2024-05-30

**Authors:** M. Padma Usha, G. Kannan, M. Ramamoorthy

**Affiliations:** ^1^ Department of Electronics and Communication Engineering B.S. Abdur Rahman Crescent Institute of Science and Technology, Vandalur, Chennai, India; ^2^ Department of Artificial Intelligence and Machine Learning Saveetha School of Engineering SIMATS, Chennai, 600124, India

## Abstract

The most common and aggressive tumor is brain malignancy, which has a short life span in the fourth grade of the disease. As a result, the medical plan may be a crucial step toward improving the well-being of a patient. Both diagnosis and therapy are part of the medical plan. Brain tumors are commonly imaged with magnetic resonance imaging (MRI), positron emission tomography (PET), and computed tomography (CT). In this paper, multimodal fused imaging with classification and segmentation for brain tumors was proposed using the deep learning method. The MRI and CT brain tumor images of the same slices (308 slices of meningioma and sarcoma) are combined using three different types of pixel-level fusion methods. The presence/absence of a tumor is classified using the proposed Tumnet technique, and the tumor area is found accordingly. In the other case, Tumnet is also applied for single-modal MRI/CT (561 image slices) for classification. The proposed Tumnet was modeled with 5 convolutional layers, 3 pooling layers with ReLU activation function, and 3 fully connected layers. The first-order statistical fusion metrics for an average method of MRI-CT images are obtained as SSIM tissue at 83%, SSIM bone at 84%, accuracy at 90%, sensitivity at 96%, and specificity at 95%, and the second-order statistical fusion metrics are obtained as the standard deviation of fused images at 79% and entropy at 0.99. The entropy value confirms the presence of additional features in the fused image. The proposed Tumnet yields a sensitivity of 96%, an accuracy of 98%, a specificity of 99%, normalized values of the mean of 0.75, a standard deviation of 0.4, a variance of 0.16, and an entropy of 0.90.

## 1. Introduction

A tumor is an unusual mass detected inside or on the brain. A tumor is a solid or fluid-filled mass of aberrant tissues. The tumor is also known as a neoplasm. According to the global cancer registered data, cancer cases in both sexes account for approximately 18,000,000, with around 20,000 instances of brain tumors. Very high HDI (human development index) regions had the maximum occurrence (102,260 cases, or 34.4%) and mortality (77,815 cases, or 32.3%) [1]. There are various approaches to imaging which are MRI, PET, and CT to diagnose the tumor's position and size. MRI is a technique that is noninvasive and produces comprehensive 3D anatomy images [2]. CT scan is another imaging technique that provides tumor information in a few seconds. Apart from this, PET is a functional imaging technique [3]. The image fusion method is stated as collecting all of the necessary data from several images and fusing them into a single fused image. More informative data will be obtained from the single fused image than from any of the input images, and it contains all the mandatory data [4]. Image fusion's objective is not just to minimize the amount of information but also to create images that are better appropriate and suited for understanding humans and machines. Image fusion is useful in medical imaging applications because it improves radiologists' detection of abnormalities in CT and MR brain images [5]. Image fusion will provide fused pictures that are more insightful than the separate input images, which makes them more appropriate for classification problems [6]. It decreases the volume of data, holds significant features, removes artifacts, and provides an output image that will be more suitable for interpretation. Image fusion can be broadly categorized as follows:

- Multimodal image fusion
- Multiview image fusion
- Multifocus image fusion
- Multitemporal image fusion

Multimodal is obtaining different imaging sensors and is used for medical diagnosis and security. A multiview is a single sensor image from different viewpoints. Multiple focal lengths of imaging equipment are used to capture multifocus images. Multitemporal refers to pictures taken at various intervals of time. There are various stages for grading image fusion processes. Multimodal fused images are employed in biomedical processing among the four categories of fusion techniques based on image acquisition. This is due to the integration of multiple pieces of information into a single image, an essential requirement for physicians to conduct in-depth analysis and proceed with further assessments [7].

The three types of fusion are feature-level fusion, pixel-level fusion [5], and decision-level fusion. Pixel-level picture fusion is thought to be the simplest and most successful method for analyzing it [8]. Unlike alternative approaches, pixel-level image fusion generates a combined image that is richer in information for both computer processing and visual perception. This is achieved through the direct integration of the original information from the source images [9]. In contrast to alternative methods, pixel-level image fusion directly integrates the inherent details from the source images to generate a fused image that is more comprehensive in terms of data for both computer processing and human vision. In these approaches, the resulting fused image incorporates either the maximum, minimum, or average values of corresponding pixels from the two input images. Feature-level fusion extracts features such as edges and textures and then fuses these supplementary features. In decision-level fusion, a decision is obtained from the source images through certain criteria and then the information from the source images is fused [9]. Among these techniques, pixel-level fusion is a simple technique, feature-level removes redundancy, and decision-level is a robust technique.

### 1.1. Image Segmentation Methods

Image segmentation techniques can be classified according to segmentation methods and their processing that is required to reach the objective of extracting features. They are

- Simple threshold method
- Edge detection-based segmentation
- Region growing and splitting technique
- Cluster model
- Watershed segmentation
- Artificial neural network-based segmentation

Among the other methods of image segmentation, segmentation based on artificial intelligence (AI) improves accuracy comparatively and also saves time. Artificial neural networks (ANNs) are among the most dominant AI techniques available, which can categorize and quantify lesions with pinpoint accuracy as well as mimic the clinical evaluation for a given problem [8].

An artificial neural network separates the defective regions of a picture by pixel-by-pixel processing [10]. After statistical features are extracted from the problematic regions, a supervised algorithm grades the image [11]. AI systems are thought to have a significant interest in the field of medical diagnosis using machine learning and image processing [12].

The most common application of artificial intelligence in medical image classification and recognition is artificial neural network approaches [13]. A significant method for the effective identification of brain tumors is the artificial neural network. Steps are taken in this work to detect brain tumors accurately [10]. Although other methods of segmentation have their own merits and demerits, the convolutional neural network method is based on decision-making by learning from the given set of images [11].

Our work's contribution is as follows:

- We propose a simplified CNN architecture and Tumnet model with a minimum number of layers in convolution, pooling, and fully connected stages compared with other CNN architectures
- Small-size kernel (3 × 3) is considered for all the layer operations
- Complexity in the number of layers is minimized with 5 convolutional layers, 3 pooling layers with ReLU activation function, and 3 fully connected layers
- We conduct a simulation of brain tumor images with meningioma and sarcoma images of the same slices by fusing the images, classification, and segmentation of the tumor area. This process can be implemented to find out postoperative tumor residual tumor cells
- Tumnet is also implemented for single-modal MRI and CT image slices to classify and extract tumor areas

## 2. Related Work

Extensive literature was made on the fusion, classification, and segmentation of brain tumor images. A detailed analysis was performed on various techniques of image fusion, segmentation, and classification. Liu et al. [12] provided a thorough analysis of recent advances in deep learning-based pixel-level image fusion techniques. They discuss the current state-of-the-art approaches, including single-shot and multiscale fusion methods, and analyze their benefits and drawbacks. Altaf [14] proposed a method for accurately delineating the gross tumor volume in brain gliomas using CT-MRI image fusion. The author presents a framework that integrates different image processing techniques, including registration, segmentation, and fusion, to achieve better tumor volume estimation. Selvakumar et al. [15] proposed a method for neoplastic segmentation and area calculation using fuzzy C-means and K-means clustering algorithms. The authors discuss the merits and demerits of each technique and show that the proposed method can achieve better segmentation results compared to other state-of-the-art methods. Rammurthy and Mahesh [16] proposed an MRI image-based deep learning method for the identification of brain tumors. The authors employ a Whale Harris Hawks optimization algorithm to make CNN learn for classification. The review provides useful insights into the cutting-edge methodologies for medical image registration and fusion [17].

Maqsood et al. [7] proposed a technique to identify brain tumors using image fusion based on CNN. Selmakuvar et al. [15] presented a narrative review of brain image segmentation methods in recent years. They discussed various techniques such as deep learning, clustering, and graph-based methods, and highlighted their advantages and disadvantages. Pereira et al. [18] developed a CNN-based method for segmenting brain tumors in MRI images. They used a small kernel of size (3 × 3) and attained 0.74 as an average dice coefficient. Ramamoorthy and Banu [19] presented a review of video enhancement techniques for medical and surveillance applications. They discussed various approaches such as contrast enhancement, noise reduction, and superresolution. Bhandari et al. [20] suggested a CNN-based segmentation of brain lesions. They used a 3D CNN architecture and attained 0.80 as the dice coefficient. Ranjbarzadeh et al. [21] developed a segmentation of brain lesions for multimodal MRI images implementing deep learning and attention mechanisms. The authors [22] proposed a thresholding-based method for medical image segmentation. They discussed various applications of thresholding techniques in medical image segmentation. Arif et al. [23] presented a technique for brain tumor identification and classification by means of biologically inspired orthogonal wavelet transform and deep learning techniques. Bahadure et al. [24] proposed a method for MRI-based detection of brain tumor and feature extraction using biologically inspired BWT and SVM. They obtained an accuracy of 95.45% for tumor detection and 91.67% for feature extraction.

Rammurthy and Mahesh [16] proposed a deep learning classifier for the identification of brain tumors utilizing MRI images. The classifier relies on Whale Harris Hawks optimization (WHHO) and can accurately classify MRI images as tumor or no tumor. Çinar and Yildirim [25] suggested a hybrid CNN architecture for brain tumor detection on MRI images. The proposed architecture combines convolutional neural networks with handcrafted features and can accurately detect brain tumors. Nayak et al. [26] suggested a classification system for brain tumors using a dense efficient-net. The proposed approach can accurately put brain tumors into distinct groups. Isin et al. [27] provide an overview of deep learning techniques for MRI-based brain tumor image segmentation. Saravanan et al. [28] proposed a CNN-based approach for the identification and classification of glioma brain lesions. Pereira et al. [29] proposed an automatic brain tumor grading approach using CNN and an assessment of quality. The proposed approach can accurately grade brain tumors based on their characteristics. Vankdothu and Hammed [30] proposed a recurrent convolutional neural network-based approach for the identification and classification of brain tumor MRI images. The technique suggested can exactly classify brain tumor MRI images into different categories. The authors proposed a multilevel CNN model for brain tumor classification in IoT healthcare systems [31].

The literature review highlights the increasing popularity of deep learning-based approaches for the detection and classification of tumors in MRI brain images. Despite these benefits, there are still challenges that need to be addressed, such as the increase in accuracy of detection, classification, and complexity in the deep learning model. The proposed system incorporates a pixel-level fusion technique for multimodal images and utilizes a simple thresholding technique for segmentation. A CNN model with a small kernel and minimal layers is utilized for the categorization of brain tumors. This model as a whole improves the accuracy of classification.

## 3. Materials and Methods

The proposed approach is divided into two sections. Preprocessing comes first, followed by the implementation of the proposed method. As an input, brain MRI and CT imaging datasets are employed. Implementation includes tumor and nontumor images. Images must be transformed to .jpg format before being used in the MATLAB environment.

### 3.1. Preprocessing

The downloadable images of the brain and its lesion can be found at http://www.med.harvard.edu/AANLIB/home.html [32] and https://www.kaggle.com/datasets/navoneel/brain-mri-images-for-brain-tumor-detection?resource=download [33]. The file format and spatial resolution of downloaded images are 256 × 256 gif. As a preprocessing, 256 × 256 images are resized to 227 × 227 as the Tumnet model was designed to process only 227 × 227 images, and the gif format is converted to jpg images for further processing. Meningioma and sarcoma types of brain tumors are taken from the database. Out of 170 sets of meningioma and sarcoma together available in the database, 154 sets of MR-CT combination (70 sets of meningioma and 84 sets of sarcoma) are considered multimodal slices from the MedHarvard database, and 561 single-modal slices (280 MRI meningioma and 281 CT sarcoma images) are considered for the dataset from the Kaggle database. This accounts for a total of 869 slices of images for further processing. Our primary concern in preprocessing here is the removal of salt-and-pepper noise, and the downloaded images from this specified database are already preprocessed and void of salt-and-pepper noise. So, the only preprocessing stage includes resizing images to 227 × 227. From the database, 170 sets of meningioma and sarcoma brain tumors were extracted. Among these, 154 sets (70 meningiomas and 84 sarcomas) were identified as multimodal slices, consisting of both MR and CT images. Additionally, 561 single-modal slices were included in the dataset, comprising 280 MRI images of meningioma and 281 CT images of sarcoma. In total, there are 869 image slices available for further processing. Training and validation images are 70% and 30%, respectively.

### 3.2. Fusion Using the Averaging Method

**Figure 1:**
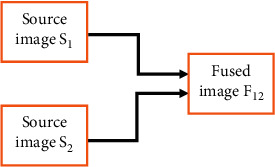
Fusion structure.

The images can be fused using the averaging method. This method takes up the two images, and the resultant images will have the average pixels of both images [34]. The pixels of each image will be considered and added, and they will be divided by the quantity of the images utilized. All the pixels in the images will continue this method, and the output fused image will be obtained. The fusion structure is depicted in Figure 1.

(1) $$\begin{array}{c} \text{Consider }N\text{ source images},\text{S}=\left\{Si,i=1,------N\right\}, \end{array}$$

(2) $$\begin{array}{c} M\text{ fusion structures},T=\left\{T_{i},i=1-------M\right\}. \end{array}$$

The fused image is stated by

(3) $$\begin{array}{c} F=\left\{Ti\left(S\right),i=1,---------M\right\}=\left\{F_{ij},i=1-----------M,j=1------------Ki\right\}. \end{array}$$

where *Ki* is an image fusion algorithm to result in infused images from *N* source images and *M* fusion structures.

The mathematical equation of image fusion is given by

(4) $$\begin{array}{c} \text{MC}1\left(x,y\right)=\frac{M1\left(x,y\right)+C1\left(x,y\right)}{2}, \end{array}$$

where MC1(*x*,*y*) is the fused MRI and CT images.

The image averaging method considers the corresponding pixels from both MRI and CT images [35] and is fused by considering the average of those pixels [36]. This method of pixel-wise image fusion carries the dominant features from a couple of MRI and CT images [8].

### 3.3. Image Segmentation with Deep Learning

**Figure 2:**
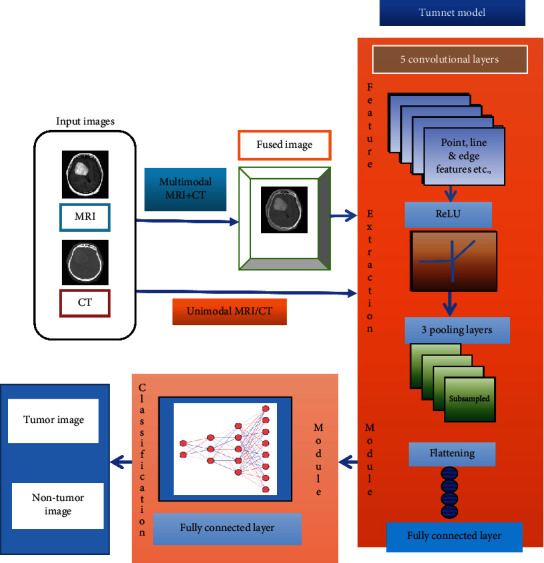
Structure of brain tumor detection model.

**Figure 3:**
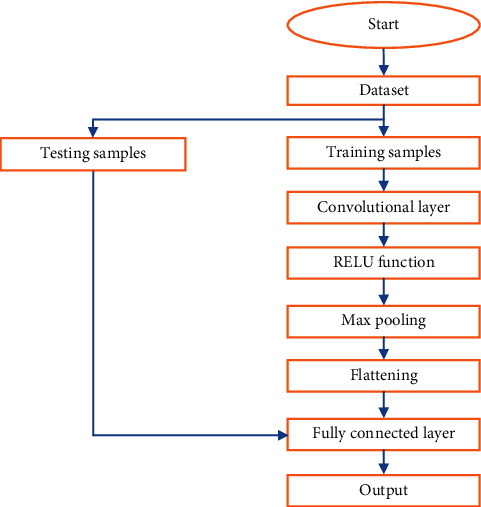
Workflow of the Tumnet model.

**Figure 4:**
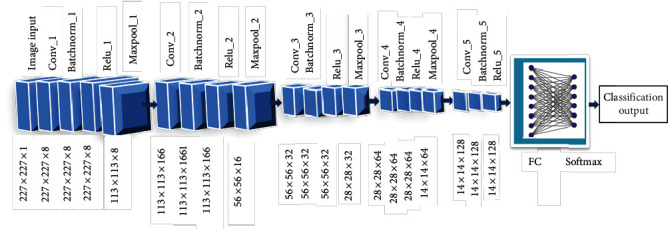
Structure of the Tumnet model.

Because it allows us to interpret the image content, image segmentation is an important aspect of computer vision and image processing [27]. It can be used for image reduction, scene interpretation, and finding objects in medical images and satellite images, among others. Many image segmentation methods have been created over time, and while several picture segmentation techniques have been developed over time, deep learning for computer vision has allowed for the evolution of numerous image segmentation deep learning models [23]. Recurrent neural networks, convolutional neural networks, deep belief networks, and multilayer perceptron (MLP) are examples of deep learning techniques [24]. CNN could be a feed-forward neural network that is usually accustomed to analyzing visual pictures by processing information with a grid-like topology. It is additionally called a Tumnet. CNN is employed to perceive a neural network which is conceived as the human visual cortex and classify objects in a picture. Figures 2 and 3 represent a block diagram of the proposed model and its corresponding flowchart [37]. In the proposed model, novelty has been brought about by combining fusion and segmentation techniques. Input brain tumor images are initially preprocessed to make them compatible with the architecture used and fused by using the image architecture for feature extraction, and image classification is performed by a fully connected layer [38]. If the tumor is present, then the tumor region is extracted and its area is calculated. The Tumnet method, which involves feature extraction and classification [25], is illustrated in Figure 3. The intricate architecture of the Tumnet model is seen in Figure 4. The Tumnet algorithm helps to extract information from images by using the ideal number of hidden layers [26]. Convolution, ReLU, pooling, and fully linked layers are the Tumnet model layers. The layer parameters and measurements are shown in Table 1 [28].

#### 3.3.1. Convolution Layer

Convolution is a process in image processing from which features can be extracted. For example, simple low pass filter, high pass filter, and image segmentation operations involve convolution [39].  Table 2 displays the sample convolution kernels to extract the features. It is clear from these operations that extraction of features requires convolution operation concerning images. Convolution operation goes on as

(5) $$\begin{array}{c} Z=X*f, \end{array}$$

where *X* is the input image, *f* is the filter, and *Z* is the filtered image.

Based on the number of filters and layers, CNN obtain the features from the given image in which the object has to be identified.

In general, convolution operation is defined mathematically as

(6) $$\begin{array}{c} g\left(x,y\right)=\underset{k,l\in w}{\sum }\sum W\left(k,l\right)f\left(x-k,y-l\right), \end{array}$$

where *f*(*x*, *y*) is the image input and *g*(*x*, *y*) is the image output.

The kernel chosen for convolution operation is 3 × 3 which makes the Tumnet model an optimum model to be implemented [12].

#### 3.3.2. Pooling Layer

The pooling layer is derived after the convolutional layer, which subsamples the pixels to reduce computational effort without impacting the individual properties of the activation maps. The pooling layer's purpose is to combine related characteristics into one [40]. For subsampling, the minimum image rate, called the Nyquist rate, must be followed.

(7) $$\begin{array}{c} f_{\text{s}}\geq 2f_{\max}, \end{array}$$

where *f*_*s*_ is the sampling frequency and *f*_max_ is the maximum sampling frequency.

The pooling layer has three major types, namely, maximum pooling, minimum pooling, and average pooling. In this technique, maximum pooling is implemented to bring dominant features from the original image. In the pooling layer, the filter size is 2 × 2 and the stride length is 2. The dimensions of the output received after a pooling layer for an activation map with size *n*_*h*_ × *n*_*w*_ × *n*_*c*_ are

(8) $$\begin{array}{c} \frac{\left(n_{h}-f+1\right)}{s}*\frac{\left(n_{w}-f+1\right)}{s}*n_{c}, \end{array}$$

where *n*_*h*_ is the activation map height, *n*_*w*_ is the activation map width, *n*_*c*_ is the number of channels in the activation map, *f* is the filter size, and *s* is the length of the stride

#### 3.3.3. ReLU Layer

The following formula applies to the rectified linear unit (ReLU): *f*(*x*) = max (0, *x*), which has become popular in recent years, as well as the more traditional sigmoids, which are nonlinear functions used in neural networks.

(9) $$\begin{array}{c} R\left(x\right)=\left\{\begin{array}{cc} x & x\geq 0,\\ 0 & \text{otherwise}, \end{array}\right. \end{array}$$

- the result equals the *x* portion of the “max” function for all *x* ≥ 0
- the result equals the 0 portions of the “max” function for all *x* < 0

Because the presence of anomalies in medical images is nonlinear, this layer is in charge of nonlinear data conversion [41]. To avoid overfitting, some of the neurons are dropped out which is a regularization method of avoiding overfitting of data. The applied dropout value is 0.6.

#### 3.3.4. Flattening Layer

**Figure 5:**
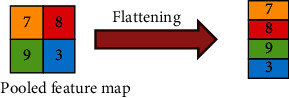
Flattening of pixels.

Flattening is a technique for converting a pooled feature map's 2D array into a long single continuous linear array.  Figure 5 illustrates the function of the flattening layer.

#### 3.3.5. Fully Connected (FC) Layer

A fully connected layer is an example of a feed-forward. The fully connected levels are the network's final tiers.

**Figure 6:**
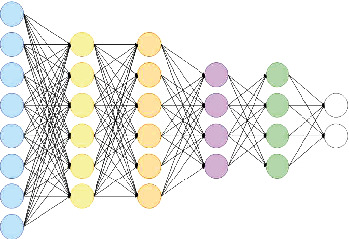
Fully connected layer.

A bias vector is added after the input has been multiplied by a weight matrix in the FC layer, as shown in Figure 6 [42]. Following the convolution and downsampling layers are one or more FC layers. Every neuron in the FC layer is linked to every neuron in the next layer. This layer collects all of the characteristics obtained by the previous layers across the image to discover the bigger patterns [43]. The characteristics are combined in the final fully linked layer which is used to classify images in classification challenges. The completely linked layer has six neurons in the softmax layer.

#### 3.3.6. Activation Function Analysis

**Figure 7:**
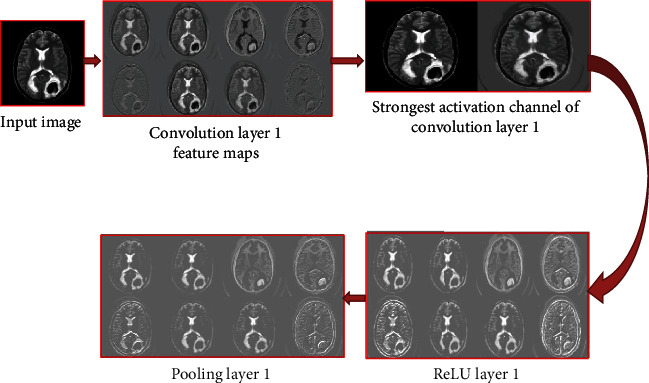
Feature maps of stage 1 of the Tumnet model.

**Figure 8:**
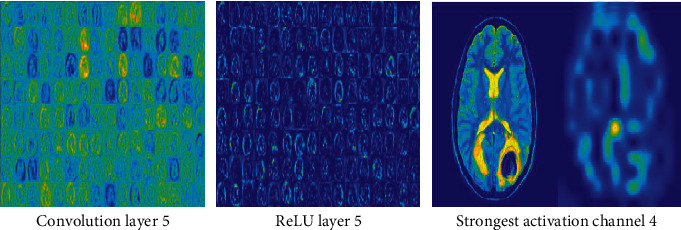
Feature maps of convolution layer 5, ReLU layer 5, and strongest activation channel 4.

Figure 7 illustrates feature maps of stage 1 of the Tumnet model, in which feature maps of pooling layer 1, convolution layer 1, and ReLU layer 1 were displayed. Also, the strongest activation channel of convolution layer 1 has been compared with the input image slice.  Figure 8 depicts the activation function outputs of convolution layer 5 and ReLU layer 5. Also, the strongest activation channel of layer 4 has been shown. Channels in the deeper layer learn complex elements like fissures and gyri, while channels in the early layers learn only simple features like color and boundaries [44].

Strong positive activations are represented by white pixels, and strong negative activations are represented by black pixels [45]. A colormap has been assigned for the feature maps to enhance the visibility of the features. Feature map regions that are gray do not activate as strongly from the image input [46]. A white pixel in a feature map represents that the input image features are carried out in the resulting feature map effectively. The figure represents the strongest activation channel of convolution layer 4. From the feature map, it is clear that the tumor part is activated well at the deeper convolution layer 4 which indicates that by implementing this architecture, it can achieve strong activations at this layer. The pseudocode of the mode is depicted in Pseudocode 1.

## 4. Experimental Outcomes

This work demonstrates the results of the segmented image of a brain lesion from the fused MRI and CT images of size 227 × 227 pixels (images downloaded from http://www.medharvard.edu) [47]. The experiment was conducted in the MATLAB 2019a version on 8 GB RAM and a 64-bit operating system. Initially, input images are resized from 256 × 256 to 227 × 227 as required by the architecture. Then, the images are fused by using pixel-level fusion, namely, the averaging method. This method carries dominant features from the original image. Image fusion is performed in brain tumors which is helpful for neurophysicians while giving treatment for radiotherapy or postoperative radiotherapy [48]. Following that, the fused pictures are trained using the CNN approach of the Tumnet model (which learns features from the dataset already provided), which includes the processes of convolution, pooling, and feature extraction (brain tumor component), with an input layer, a hidden layer, and an output layer. If a tumor exists, the size of the tumor component is retrieved from brain imaging by using a simple threshold method. Structural similarity (SSIM) for image fusion, as well as sensitivity, specificity, entropy, standard deviation, and variance, are calculated as performance measures.

- Structural similarity (image fusion)

It is a measure of structural similarities between two images in which one image is a reference image and the other one is compared with this image [49]. SSIM is found by

(10) $$\begin{array}{c} \text{SSIM }\left(x,y\right)=\frac{\left(2xy+C_{1}\right)\left(2Sxy+C_{2}\right)}{\left(x^{2}+y^{2}+C_{1}\right)\left(S_{x}^{2}+S_{y}^{2}+C_{2}\right)}, \end{array}$$

where *C*_1_ and *C*_2_ are (*k*_1_*L*)^2^ and (*k*_2_*L*)^2^.

*k*_1_ and *k*_2_ are small constants of values 0.01 and 0.03.

*L* is the pixel's dynamic range (*L* is 8 for 0-255 grayscale range image).

(ii) Sensitivity

An algorithm can correctly identify a disease. The mathematical formula is given by

(11) $$\begin{array}{c} \text{Sensitivity}=\frac{\text{true positive}\left(\text{TP}\right)}{\text{true positive }\left(\text{TP}\right)+\text{false negative }\left(\text{FN}\right)}. \end{array}$$

(iii) Specificity

An algorithm can correctly identify a disease that is not there.

(12) $$\begin{array}{c} \text{Specificity}=\frac{\text{true negative }\left(\text{TN}\right)}{\text{true negative }\left(\text{TN}\right)+\text{false positive }\left(\text{FP}\right)}, \end{array}$$

where TP is the true positive, segmented pixels appropriately stated as positive; FP is the false positive, segmented pixels inaccurately stated as positive; TN is the true negative, segmented pixels appropriately stated as negative; and FN is the false negative, segmented pixels inaccurately stated as negative.

(iv) Entropy

The information content of an image is measured by entropy. It describes how much randomness or ambiguity is present in an image. The higher the quality of a photograph, the more details it holds. The more the entropy, the more detailed the image will be.

(13) $$\begin{array}{c} E\left(I\right)=\overset{L-1}{\underset{0}{\sum }}P\left(K\right)\log_{2}P\left(K\right), \end{array}$$

where *I* is the original image, *P*(*K*) is the probability of the value *k* appearing in image *I*, and *L* is the number of various gray levels.

(v) Standard deviation

The standard deviation describes how far the values in a dataset depart from the average. One technique to assess contrast is to provide the pixel value standard deviation in an image.

(vi) Variance

A random variable's variance indicates how far it diverges from its mean value. The variance is the average of the squares of the discrepancies between the individual value and the expected value. This implies that it is always in the affirmative.

### 4.1. Discussion

**Figure 9:**
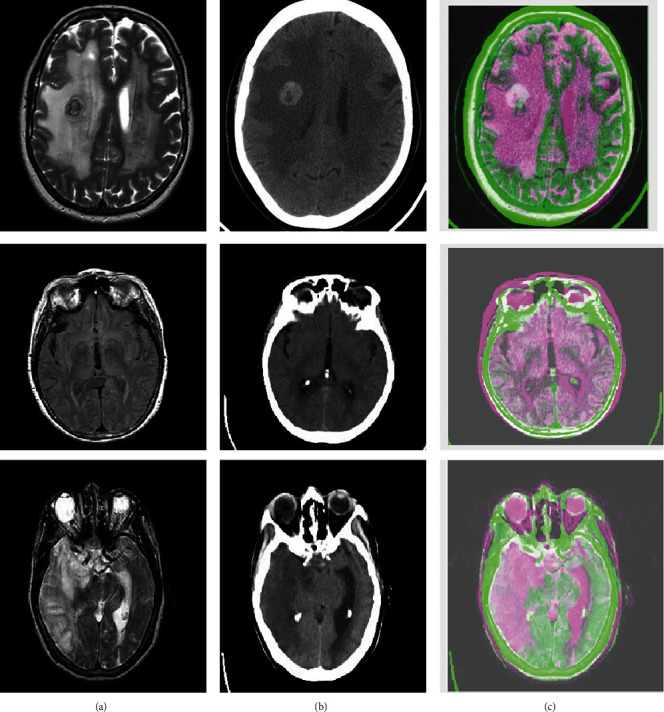
MRI and CT Brain of size 256 × 256 pixels and its fused images. (a) Input-MRI image. (b) Input-CT image. (c) Fused MRI-CT image by using the average method.

Figure 9 depicts a sample of input MRI and CT images in an axial slice, with the MRI revealing a tumor as well as the outline of gray and white matter [36]. The fused image depicts a combination of prominent MRI and CT features as a consequence of the pixel-level fusion averaging approach. This approach is deemed simple because it works with pixels directly. Other pixel-level fusion approaches, such as the maximum and minimum methods, are contrasted with this fusion method. This is called fusion at the feature level as stated by and classification by using advanced classification techniques such as CNN combined with small kernel concept. This seems to be the novelty of this proposed method. This colearning of a different set of features from various modals can be an added advantage to the convolutional neural network which in turn extracts features from the fused multimodal image. As far as carcinoma is concerned, an accurate diagnosis is so vital that even one residual cell can multiply into many [50]. This can be achieved by implementing multimodal images for feature extraction, followed by multimodal tumor classification (presence/absence of a tumor).

This kind of fusion-based CNN can be applied for patients who are taking both the MRI and CT scan as well as those patients who are undergoing radiotherapy after the operation of the tumor. During the treatment of radiotherapy, fusion-based classification is important in detecting any tumor cells.  Figure 8 shows the extracted tumor outline from the fused image with the implementation of the CNN method. This method is implemented with the simple threshold method, with an object solidity value greater than 0.7 and object areas greater than 100 pixels considered to be the tumor region [21]. Only the two parameters mentioned above can be changed in a simple threshold technique to extract tumor outlines.

**Figure 10:**
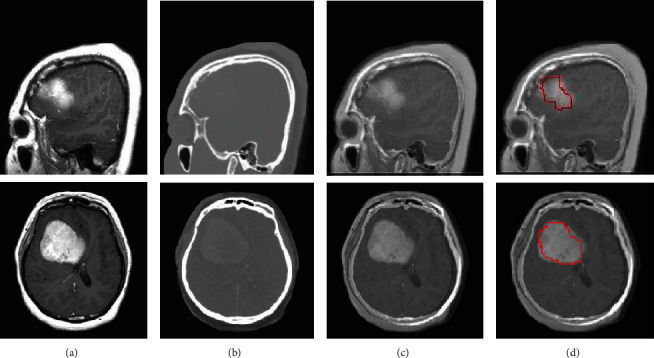
Extraction of brain tumor images. (a) Input-MRI image. (b) Input-CT image. (c) Fused MRI-CT image. (d) Tumor outline.

Although the goal of brain lesion detection is to obtain active tumorous tissue and tumor regions that have spread, the localization and detection of active tumorous tissue were the focus of this study [51]. The tumor's detected region is then excised, and the tumor area in mm^2^ is determined, as shown in Table 3. The extracted tumor image is shown in Figure 10. Various performance metrics for image fusion and image segmentation techniques for tumor images were calculated [52].  Table 4 depicts the fusion technique's performance metrics derived by combining the averaging approach, the minimum pixel-level fusion method, and the maximum pixel-level fusion method, as well as a basic threshold and CNN. When both procedures are compared, the averaging method produces higher values for both SSIM tissue and SSIM bone. SSIM tissue is determined by comparing the MRI input picture with the fused image, whereas SSIM bone is determined by comparing the input CT image with the fused image. This metric conveys how far the tissue and bone structures are carried over to the fused image. A higher value of SSIM tissue and bone provides more details in the merged image. Tables 5, 6, and 7 show the analysis of fusion metrics for average, maximum, and minimum methods. It also illustrates the minimum and maximum range of performance metric values for the three fusion methods, in which the comparatively average method outperforms well. Apart from the gold standard metric SSIM, parameters like the fused images, standard deviation, and entropy are also determined, which are also shown in Table 7. Standard deviation conveys the deviation of the pixel value from the mean value, and entropy ranges between 1 and 8 for a 0 to 255 grayscale fused image.

Table 8 illustrates the brain tumor image segmentation metric comparison of IFST techniques for the same set of images. Parameters like accuracy, sensitivity, specificity, standard deviation, and entropy were calculated, and the accuracy value ranges from 60% to 90% for the IFST method. The next important metrics are specificity and sensitivity, which are high for the proposed method and are around 96% and 95% on average, respectively, as shown in Table 8. This is because pixel average is carried out at the output, whereas in the other methods of pixel-level fusion, there is a chance of a missing tumor due to the minimum pixel value or maximum pixel value at which the tumor may or may not be present.

The fusion approach is the first step in the simulation process. This pixel-level fusion approach is used in this technique. Initially, the average fusion technique is applied in which fusion metrics are obtained for 154 sets of tumor images. Fusion metrics displayed were SSIM bone, SSIM tissue, entropy of fused image, entropy of tumor image, mean of fused image, mean of tumor image, standard deviation of fused image, standard deviation of tumor image, variance, sensitivity, specificity, and accuracy. SSIM tissue ranges from 44% to 99%, and SSIM bone ranges from 61% to 98%, whereas the maximum method varies from 1% to 98% and the minimum method ranges from 1% to 98%. Although there is not much difference between the maximum value among the three methods, it is to be noted keenly that the minimum value of the maximum and minimum methods falls to a very low value of 1. Apart from second-order advanced statistical parameters, first-order statistical parameters are also obtained by using the three methods. The normalized value of sensitivity, specificity, and accuracy reaches its maximum value at 1. Among these parameters, SSIM tissue and bone convey to us the true scenario of the proposed algorithm performance.  Table 7 conveys the average of fusion metrics for 154 sets of tumor images from five patients who suffered from meningioma and sarcoma cancers, in which sarcoma is a metastatic tumor [48]. T1-weighted, T2-weighted, and proton-density sequences of brain tumor images are available (PD) [42]. Images are sliced axially, sagittally, and coronally, in which axial slices are usually preferred since they contain most of the parts of the brain. Images belong to a different set of patients having the abovementioned abnormalities in their brain images. This implies that, for a wide variety of cases, our proposed algorithm suits well on average. Fusion-based convolutional modal network is preferred for specific cases such as those patients who are asked to take both MRI and CT after radiotherapy treatment [53].

Considering the average values of the following methods, such as Average-ST (simple threshold), Max-ST, and Min-ST, this implies that for a given set of 154 tumor images, this method holds good. This can be proved for more images also. The Tumnet model is implemented along with the average image fusion method. Tables 8 and 3 illustrate that the proposed method has a higher range of performance on average for the available tumor images. Performance metrics have been illustrated for both fusion as well as segmentation. The tumor area is calculated by using the formula of $\sqrt{T}*0.264$, where *T* is the segmented tumor in pixels, and this formula gives the value in mm^2^.

**Figure 11:**
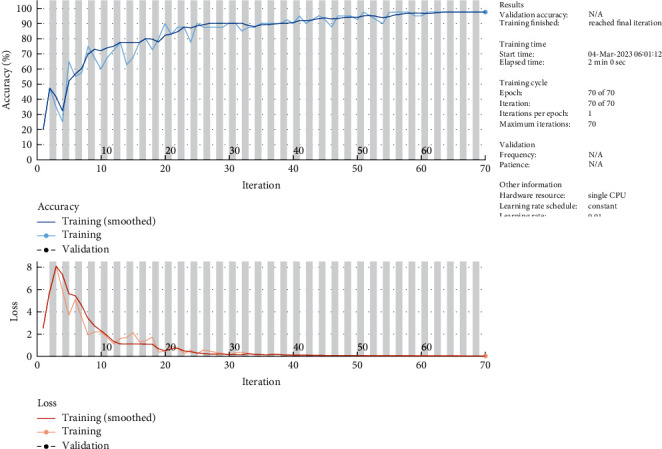
Classification accuracy and loss function of the Tumnet model.

It is observed that classification accuracy increases to 98% and loss function decreases to almost 0 for the given set of images, as shown in Figure 11. Comparative analysis with the latest references for classification accuracy is shown in Table 9.

Tumnet, being the proposed model, shows the highest performance of 96% in testing accuracy parameters for the Kaggle dataset [33] which is explicitly shown in Table 10. Hence, the Tumnet model outperforms the other existing models such as VGG-19, Alexnet, GoogLeNet, and ConvNet for the dataset considered for classification. Among these models, VGG-19 and Alexnet show low-level variation with the proposed model which has 94% and 82%, respectively, whereas GoogLeNet and ConvNet indicate a high level of variation which shows 78% and 67%, respectively. Thus, the Tumnet model proves its robustness for different datasets.

Tumnet, the proposed model, performs to obtain an accuracy of 98% for multimodal images of MRI, CT, and fused MRI-CT images. A few methods shown in Table 11 outperform the Tumnet model, but they are limited to single-modality MRI images, whereas the Tumnet model shows its high potential for diversified images. Tumnet's strength lies in its ability to handle multimodal data, achieving a notable 98% accuracy. The results underscore the importance of model architecture, dataset characteristics, and multimodal approaches in achieving high accuracy in brain tumor detection.

## 5. Conclusion

This research work proposes a Tumnet deep learning model for the categorization of brain tumors from MRI, CT, and fused MRI-CT slices. The model comprises 11 layers, including convolution, pooling, and activation layers, with a smaller kernel size of 3 × 3 for the convolution layer. The proposed system was used with the MedHarvard database of different tumors, including meningioma and sarcoma. The performance of the 3 × 3 kernel architecture was compared with larger filter architectures. The results of the research showed that the proposed method achieved high accuracy, sensitivity, and specificity in detecting brain tumors in both multimodal and single-modal MRI/CT images. Hence, the proposed approach possesses the capacity to assist physicians in accurately diagnosing and treating brain tumors. In the future, the potential of the Tumnet model can be extended to other imaging databases by applying the 3 × 3 kernel to other standard models, which may enable accurate classification and decision support for oncologists.

## Figures and Tables

**Pseudocode 1:**
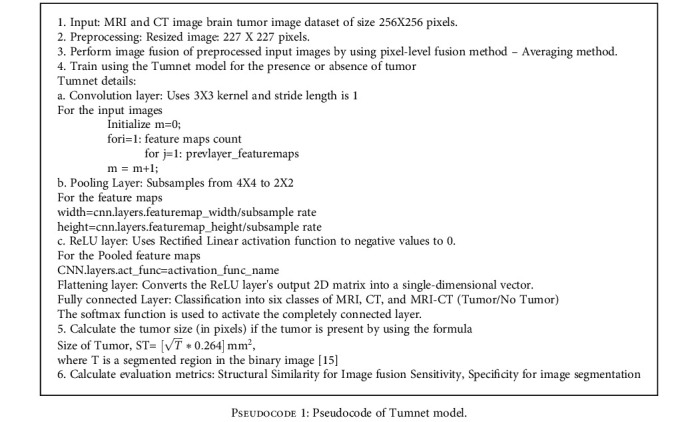
Pseudocode of Tumnet model.

**Table 1:** Learnable parameters of the Tumnet model.

S. no.	Layer name	Layer type	Size of activations	Size of learnables	Total learned
1	Image I/P 227 × 227 × 1 with “zero center normalization”	Image input	227 × 227 × 1	—	0
2	Conv_1 8 3 × 3 × 1 Convolutions stride [1 1] with padding “same”	Conv layer	227 × 227 × 8	Weight 3 × 3 × 1 × 8 Biases 1 × 1 × 8	80
3	Batch-norm_1 8 channel batch normalization	Batch normalization	227 × 227 × 8	Off-set 1 × 1 × 8 Scale size 1 × 1 × 8	16
4	ReLU_1 ReLU layer	ReLU layer	227 × 227 × 8	—	0
5	Maxpool_1 2 × 2 max pooling stride [2 2] with padding [0,0,0,0]	Maximum pooling	113 × 113 × 8	—	0
6	Conv-2 16 3 × 3 × 8 convolution stride [1 1] with padding “same”	Conv layer	113 × 113 × 16	Weight 3 × 3 × 8 × 16 Biases 1 × 1 × 16	1168
7	Batch-norm_2 Batch normalization and 16 channels	Batch Normal form	113 × 113 × 16	Off-set 1 × 1 × 16 Scale size 1 × 1 × 16	32
8	ReLU-2 ReLU	ReLU layer	113 × 113 × 16	—	0
9	Maxpool_2 2 × 2 max pooling stride [2 2] with padding [0,0,0,0]	Maximum pooling	56 × 56 × 16	—	0
10	Conv-3 32 3 × 3 × 16 convolution stride [1 1] with padding “same”	Conv layer	56 × 56 × 32	Weight 3 × 3 × 16 × 32 Biases 1 × 1 × 32	4640
11	Batch-norm_3 Batch normalization and 32 channels	Batch Normal form	56 × 56 × 32	Off-set 1 × 1 × 32 Scale size 1 × 1 × 32	64
12	ReLU-3 ReLU	ReLU layer	56 × 56 × 32	—	0
13	Maxpool_3 2 × 2 max pooling stride [2 2] with padding [0,0,0,0]	Maximum pooling	28 × 28 × 32	—	0
14	Conv-4 64 3 × 3 × 32 convolution stride [1 1] with padding “same”	Conv layer	28 × 28 × 64	Weight 3 × 3 × 32 × 64 Biases 1 × 1 × 64	18496
15	Batch-norm_4 Batch normalization and 64 channels	Batch Normal form	28 × 28 × 64	Off-set 1 × 1 × 64 Scale size 1 × 1 × 64	128
16	ReLU-4 ReLU	ReLU layer	28 × 28 × 64	—	0
17	Maxpool_4 2 × 2 max pooling stride [2 2] with padding [0,0,0,0]	Maximum pooling	14 × 14 × 64	—	0
18	Conv-5 128 3 × 3 × 64 convolution stride [1 1] with padding “same”	Conv layer	14 × 14 × 128	Weight 3 × 3 × 64 × 128 Biases 1 × 1 × 128	73856
19	Batch-norm_5 Batch normalization and 128 channels	Batch Normal form	14 × 14 × 128	Off-set 14 × 14 × 128 Scale size 1 × 1 × 128	256
20	ReLU_5	ReLU	14 × 14 × 128	—	—
21	FC	Fully connected layer	1 × 1 × 6	Weight 6×25088 Biases 6 × 1	150,534
22	Soft maximum	Soft-max	1 × 1 × 6	—	—
23	Class-output	Classification O/P	—	—	—

**Table 2:** Filter kernels in the convolution layer.

Low pass filter	$\frac{1}{9}\left(\begin{array}{ccc} 1 & 1 & 1\\ 1 & 1 & 1\\ 1 & 1 & 1 \end{array}\right)$
High pass filter	$\left(\begin{array}{ccc} -1 & -1 & -1\\ -1 & 8 & -1\\ -1 & -1 & -1 \end{array}\right)$
Line detection	$\left(\begin{array}{ccc} -1 & -1 & -1\\ 2 & 2 & 2\\ -1 & -1 & -1 \end{array}\right)$
Vertical line detection	$\left(\begin{array}{ccc} -1 & 2 & -1\\ -1 & 2 & -1\\ -1 & 2 & -1 \end{array}\right)$
Slanted line detection (45°)	$\left(\begin{array}{ccc} -1 & -1 & 2\\ -1 & 2 & -1\\ 2 & -1 & -1 \end{array}\right)$
Slanted line detection (-45°)	$\left(\begin{array}{ccc} 2 & -1 & -1\\ -1 & 2 & -1\\ -1 & -1 & 2 \end{array}\right)$

**Table 3:** Tumor area in mm^2^.

Method	Tumor area (mm^2^)					
	Set 1	Set 2	Set 3	Set 4	Set 5	Set 6
Average-ST	4.00	4.80	5.00	5.00	3.58	3.55
Max-ST	4.25	4.20	4.3	4.20	4.02	4.02
Min-ST	5.00	5.00	5.02	4.80	4.01	4.00

**Table 4:** Fusion metrics.

Method	SSIM tissue	SSIM bone	Accuracy (%)	Sensitivity (%)	Specificity (%)	Standard deviation	Entropy
Average	82.67	83.67	90.33	96.00	95.33	78.67	4.23
Maximum	67.83	67.33	84.67	73.83	74.83	75.00	3.93
Minimum	72.83	71.00	80.50	72.33	73.00	82.50	3.78

**Table 5:** Average method analysis.

Fusion metrics	Minimum value	Maximum value
SSIM tissue (%)	44.56	99.49
SSIM bone (%)	61.32	98.59
Entropy of fused image	0.9447	1
Entropy of tumor image	0	0.9877
Variance	0	0.2475
Standard deviation of fused image	60.1181	71.4693
Standard deviation of tumor image	0	0.4974
Mean of fused image	41.05	65.9838
Mean of tumor image	0	0.4473
Sensitivity (0-1)	0.47	1
Specificity (0-1)	0.93	0.99
Accuracy (0-1)	0.88	1

**Table 6:** Maximum method analysis.

Fusion metrics	Minimum value	Maximum value
SSIM tissue (%)	1	98.45
SSIM bone (%)	56.47	98.56
Entropy of fused image	0.89	0.99
Entropy of tumor image	0.85	0.99
Variance	0.20	0.24
Standard deviation of fused image	86.93	99.25
Standard deviation of tumor image	0.4	0.49
Mean of fused image	54.94	88.53
Mean of tumor image	0.27	0.49
Sensitivity (0-1)	0.54	1
Specificity (0-1)	0.99	1
Accuracy (0-1)	0.78	0.99

**Table 7:** Minimum method analysis.

Fusion metrics	Minimum value	Maximum value
SSIM tissue (%)	1	98.56
SSIM bone (%)	46.56	98.56
Entropy of fused image	0.82	0.98
Entropy of tumor image	0.79	0.99
Variance	0.22	0.25
Standard deviation of fused image	89.98	98.11
Standard deviation of tumor	0.46	0.5
Mean of fused image	41.87	91.32
Mean of tumor image	0.30	0.51
Sensitivity (0-1)	0.60	1
Specificity (0-1)	0.98	0.99
Accuracy (0-1)	0.71	1

**Table 8:** Segmentation metrics.

Method	Accuracy (%)	Sensitivity (%)	Specificity (%)	Mean	Standard deviation	Variance	Entropy
Average-ST	90	96	99	0.75	0.4	0.16	0.90
Max-ST	65	54	75	0.41	0.4	0.16	0.7
Min-ST	78	65	78	0.51	0.5	0.25	0.77

**Table 9:** Comparative analysis for tumor classification accuracy.

Reference	Year	Deep learning model	Accuracy (%)
Proposed	2022	Tumnet model	98
[31]	2022	Resnet50	95
[30]	2022	Recurrent CNN	95
[54]	2022	Improved CNN	93
[29]	2018	Convolutional neural network (CNN)	89

**Table 10:** Comparison of testing accuracy of the Tumnet model with other existing models.

CNN model	Testing accuracy (%)
Tumnet (proposed)	96
VGG-19	94
Alexnet	82
GoogLeNet	78
ConvNet	67

**Table 11:** Comparison of Tumnet model accuracy with other existing models of MedHarvard images.

CNN model	Year	Number of samples from MedHarvard	Image modality (single modal/multimodal)	Accuracy (%)
Tumnet (proposed)	2023	869	Multimodal (MRI, CT, & MRI-CT)	98
Lightweight CNN (RP2)	2023	1665	Single modal (MRI)	99.58
23 layer CNN (RP15)	2022	884	Single modal (MRI)	100
Improved Alexnet (RP5)	2020	359	Single modal (MRI)	96.43
DWT + ICA + SVM with Gaussian RBF (RP11)	2020	226	Single modal (MRI)	98.87

## Data Availability

The multimodal brain tumor dataset such as MRI and CT has been downloaded from the MedHarvard Brain Atlas, and its website address is https://www.med.harvard.edu/aanlib/. The image file format is in .gif, and it is converted to .jpg as a preprocessing step. It has a 256 × 256 pixel size. 154 sets of tumor images of the same slices from two different patients affected with meningioma and sarcoma. Each set consists of a single MRI and CT slice. Totally, 308 slices are considered for analysis on this website. Also, 561 slices of MRI are downloaded from https://www.kaggle.com/datasets/navoneel/brain-mri-images-for-brain-tumor-detection?resource=download of http://www.kaggle.com. Totally, 869 MRI and CT individual slices are considered for analysis from the MedHarvard Brain Atlas and Kaggle websites.
